# Quantitative Determination of Andrographolide and Related Compounds in *Andrographis paniculata* Extracts and Biological Evaluation of Their Anti-Inflammatory Activity

**DOI:** 10.3390/foods8120683

**Published:** 2019-12-14

**Authors:** Emmanuelle Villedieu-Percheron, Véronique Ferreira, Joana Filomena Campos, Emilie Destandau, Chantal Pichon, Sabine Berteina-Raboin

**Affiliations:** 1 Institut de Chimie Organique et Analytique, Université d’Orléans, UMR CNRS 7311, BP 6759, 45067 Orléans CEDEX 2, France; emmanuelle.percheron@univ-orleans.fr (E.V.-P.); veronique.ferreira@univ-orleans.fr (V.F.); joana-filomena.mimoso-silva-de-campos@univ-orleans.fr (J.F.C.); emilie.destandau@univ-orleans.fr (E.D.); 2 Centre de Biophysique Moléculaire CNRS UPR 4301, Université d’Orléans, Rue Charles Sadron, F-45071 Orléans CEDEX 2, France; Chantal.PICHON@cnrs.fr

**Keywords:** andrographolide, reversed-phase liquid chromatography, quantitative analysis, method validation, anti-inflammatory activity

## Abstract

Extraction, isolation and characterization of *Andrographis paniculata* (*A.p.*) products were developed. Three natural diterpenes compounds were obtained and one was used for chemical modifications. Evaluation of their inhibition of TNFα induced NFκB transcriptional activity. A rapid analytical method for the determination and quantitation of three diterpenoid lactones (andrographolide **1**, didehydroandrographolide **2**, neoandrographiside **3**) found in *A. paniculata* extracts was investigated. After some optimizations on column type and injection solvent, the separation was achieved in 9 min on a monolithic Chromolith Performance RP18e column (100 mm × 4.6 mm ID, 2 µm), with a gradient solvent system of water and methanol, UV detection at 220 nm and ELSD detection. The method was proved to be suitable for the quantitation of these three diterpenes in four different commercial *Andrographis* dietary supplements. The anti-inflammatory activities of a mixture of known composition have been evaluated showing differences in activity depending on the relative ratio of various diterpenes and also a possible synergic activity for some of them.

## 1. Introduction

**Figure 1:**
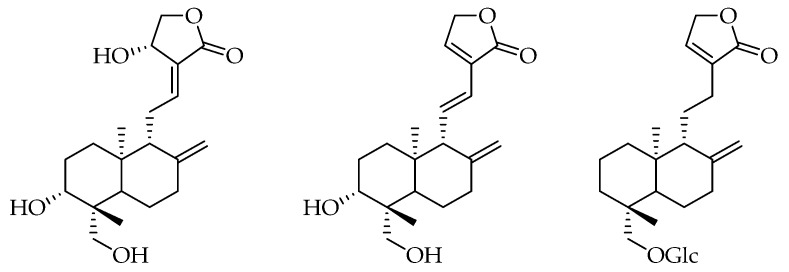
Structure of natural the compounds.

*Andrographis paniculata* (Acanthaceae) (*A.p.*) is widely used as medicinal herb in traditional medicine [1,2,3]. Diterpenoid lactones isolated from these extracts have been the subject of intensive investigations and are reported to exhibit a wide spectrum of biological activities, including antibacterial [4], anti-inflammatory [5,6,7,8], hepatoprotective [9,10,11], neuroprotective [12], cardioprotective [13,14] and anticancer properties [15,16]. Andrographolide **1** (Figure 1) has been reported to be the main active compound in the plant [17,18]. Its structure and stereochemistry have been determined over many years using chemical and spectroscopic techniques [19], and its crystal structure was characterized [20]. Other minor diterpenoid lactones also have been isolated from the plant, and their structures have been investigated [21] but their activities are not well studied. Studies about the pharmacological mechanism of its known anti-inflammatory actions have shown in 2004 that andrographolide attenuates inflammation by inhibition of NFκB activation through a covalent modification of reduced cysteine [22].

**Figure 2:**
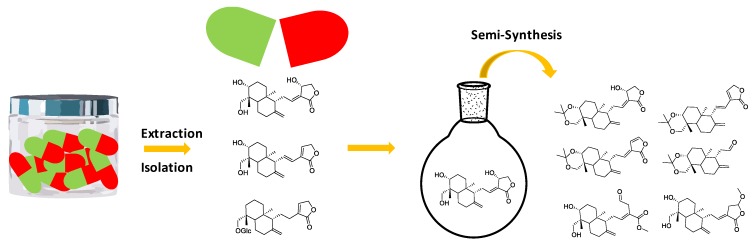
Composition of dietary supplements of natural compounds and pharmacomodulation.

Considering the numerous biological applications of these compounds, commercial sources of preparations based on different parts of the plant have been available for a few years. However, the box does report if the given contents concern the major Compound **1** or the whole family of diterpenes, and the method of preparation. It is well-known that the composition could change depending on the origin and the season of gathering and on the part of plant studied (roots, leaves, stem, etc.). Nevertheless, a variation in the ratio of Compound 1 as well as related compounds had not been investigated and could lead to a change in therapeutic values. Considering that the composition of these dietary supplements is unclear and in order to control their quality, it appeared to be important for us to be able to quantify the composition of such commercial preparations, in order to connect their chemical composition with the anti-inflammatory activities generated by these commercially available preparations (Figure 2).

HPLC is the most frequently used chromatographic technique in the laboratory setting. Some qualitative HPLC analyses have been reported in literature for these compounds [23,24]. HPTLC assays of *A.p*. extracts based on the quantification from spot UV visualizations have been described by Srivastava [25]; however, the extraction solvent influences the amount and nature of the extracted compounds and methanol leads to better extraction yields than chloroform or ethyl acetate. In addition, these methods often take long times and/or specific instrumentation. A few years ago, some groups developed a new rapid method for the determination of andrographolide in mixtures [24,26,27,28]. To complement these methods, we have developed a rapid and simple method for the separation and quantitation of andrographolide **1**, didehydroandrographolide **2** and neoandrographiside **3**. Based on the literature [23], using an Altima RP C18 column, an optimization of the elution gradient allowed us to obtain a better separation of the compounds present in the plant. Then we used a column dedicated to fast analysis of samples, the Chromolith Performance RP-18e column. This type of column allows to work at higher flow rates than with conventional columns of the same diameter without loss of efficiency or excessive increase in pressure. To assess to this work, the developed method was applied to four commercial preparations containing between 4% and 10% of andrographolides according to the labeling. Finally, the NFκB inhibition of these preparations was evaluated.

## 2. Materials and Methods

### 2.1. Chemicals and Reagents

Methanol from SDS Carlo Erba (Val de Reuil, France) and water purified (resistance < 18 MΩ) from ultra-pure water using an Elgastar UHQ II system (Elga, Antony, France) were used for the HPLC mobile phase and to prepare the stock solutions of analytes. Methanol from Sigma Aldrich (Steinheim, Germany) was used for the extraction and purification of crude extracts. Chloroform from SDS Carlo Erba (Val de Reuil, France) was used for purification of crude extracts.

### 2.2. Instrumentation and Conditions

**Figure 3:**
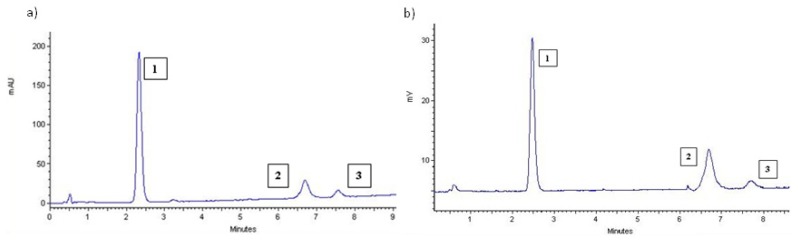
Chromatogram of the crude extract of *Andrographis* from sample A. Chromolith Performance RP-18e (100 × 4.6 mm ID, 2 µm); solvent A: water; solvent B: MeOH; gradient conditions: solvent B from 40% to 51% over 9 min, at 3 mL min, room temperature. (**a**) UV at λ = 220 nm, (**b**) ELSD.

An Agilent HP 1100 system (Waldbronn, Germany) with a 20 µL loop, coupled to a Kontron Ultra-Violet (UV) detector (Zurich, Switzerland) and piloted by EZchrome Elite workstation software was used. LC separation was performed on a Chromolith Performance RP-18e column (100 mm × 4.6 mm ID, 2 µm) provided by Merck (Darmstadt, Germany), with a gradient system containing water as solvent A and Methanol as solvent B, using solvent B from 40% to 51% over 9 min, at 3 mL·min^−1^. To avoid any peak tailing and samples precipitation, they were injected in a mixture of water:methanol of 80:20. Detection was done by UV at 220 nm, and by ELSD giving the profile shown in Figure 3 where we can see the detection of the three main compounds present in the plant: andrographolide **1**, didehydroandrographolide **2** and neoandrographiside **3.**

### 2.3. Extraction Procedure

Commercial *Andrographis* products, all in the form of tablets, were obtained from online dietary supplements shops. To protect the manufacturer’s identity, the samples were labeled with letters A–D. Extraction of diterpenoid lactones from tablets A–D was performed with a methanol ratio 15 mg·mL^−1^, and the sample was sonicated at room temperature for 60 min. The mixture was filtered through filter paper (Whatman #1) and the residue was returned to the sample vial. The above extraction procedure was repeated two more times. The combined methanol extracts were evaporated under reduced pressure to give a green powder as the crude extract.

### 2.4. Preparation of Pure Compounds

**Figure 4:**
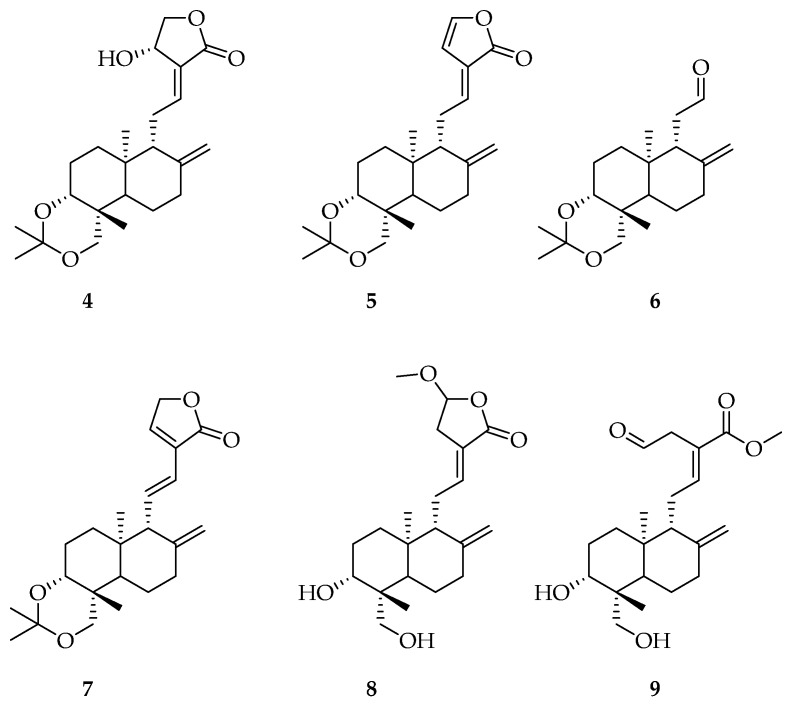
Structures of chemically modified derivatives.

**Scheme 1:**
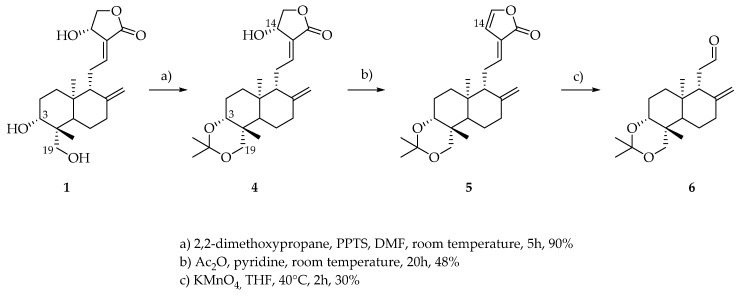
Modification of andrographolide.

External standards were obtained after purification of crude extracts from sample A by flash chromatography over silica gel (Geduran^®^ Si 60, Merck), with an elution gradient of methanol from 3% to 15% in chloroform [29]. Maximal absorbance wavelengths were 250 nm and 224 nm for Compounds **1** and **2,** respectively, whereas Compound **3** absorbed up to 220 nm. The purity of the compounds was determined by HPLC with an Altima RP C18 column and gradient system containing water as solvent A and MeOH as solvent B, using solvent B from 20% to 100% over 45 min, at 1 mL·min^−1^. Using UV and ELSD detection and washing the column for 10 min with MeOH, purity of each compound has been estimated to be superior to 99%. Further identification by NMR spectroscopy (^1^H, ^13^C, 1D, 2D) and LC-ESI-MS ([M + H]^+^, [M − H]^−^) were necessary to confirm the structures, and the results are in agreement with the literature and listed in the Results and Discussion section (Section 3.4). Semi-synthetic compounds from **4** to **7** (Figure 4) were obtained by treatment of **1** according to the procedure described in the literature [30] (Scheme 1).

**Scheme 2:**
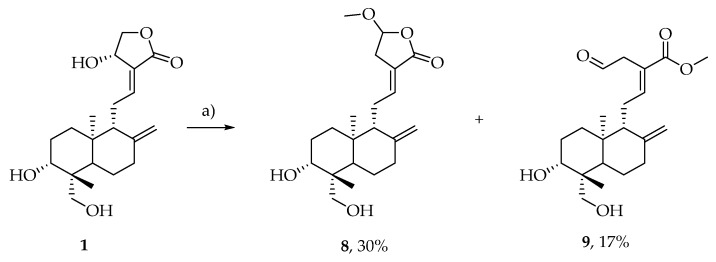
Opening of the lactone.

**Scheme 3:**
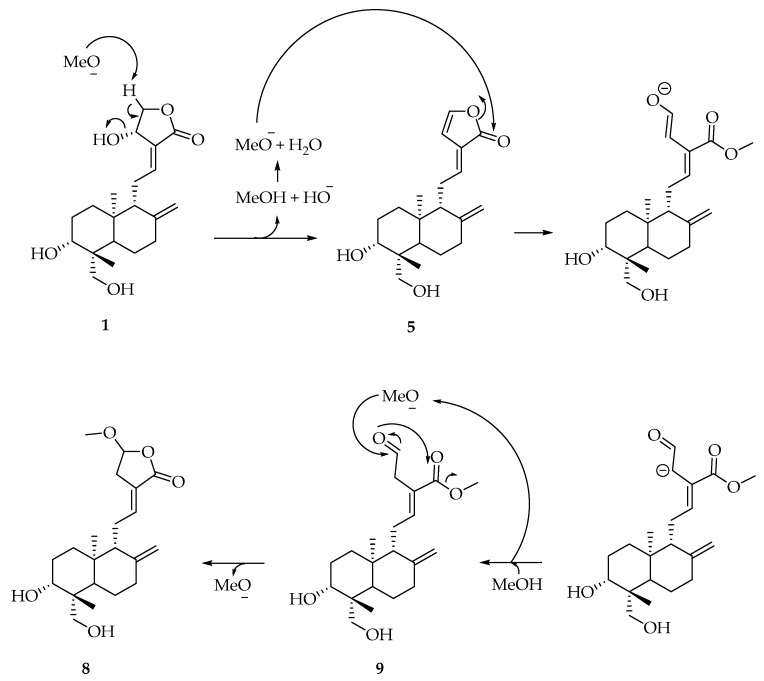
Proposed mechanism for formation of Compounds **8** and **9**.

Compound **7** came from the standard isomerization of Compound **5** and the semi-synthetic Compounds **8** and **9** were obtained after treatment of **1** with MeONa in MeOH [31] at room temperature (Scheme 2) according to the mechanism proposed in Scheme 3.

### 2.5. Standard and Sample Preparation

Stock solutions of each compound were prepared by dissolving them in methanol to obtain a 1 mg·mL^−1^ solution. Five calibration solutions were prepared by mixing the stock solution in a suitable proportion of water and methanol to have a final composition of injection solvent equal to a water:methanol of 20:80. The five concentrations were 50, 100, 125, 150 and 200 µg·mL^−1^ for Compound **1**, 50, 75, 100, 150 and 200 µg·mL^−1^ for Compound **2**, and 5, 10, 20, 40 and 50 µg·mL^−1^ for Compound **3**. Sample preparations were made according to the following procedure: About 1 mg of crude extract was accurately weighted and methanol was added to obtain a 1 mg·mL^−1^ solution. This solution was diluted with a water:methanol ratio of 20:80 to a final concentration of 0.5 mg·mL^−1^.

### 2.6. NFκB-Dependent Luciferase Activity Assay

A549/NFκB-luc cells were obtained by co-transfection of A549 cells (ATCC P/N CCL-185) with pNFκB-luc (Panomics P/N LR0051) and pHyg followed by hygromycin selection. These cells were obtained from Panomics and they maintain a chromosomal integration of a luciferase reporter gene regulated by multiple copies of NFκB response elements. They were cultured in DMEM medium supplemented with 10% FBS in presence of 100 µg/mL of hygromycin, 100 units/mL of penicillin and 100 μg/mL of penicillin and streptomycin, respectively. The NFκB-promoter luciferase reporter was used to assay the activity of NFκB activation. Stock solutions of samples were prepared in dimethyl sulfoxide (DMSO) with a concentration of 10 mg/mL. A549/NFκB-luc cells were incubated in with samples or its solvent and stimulated with TNFα (10 ng/mL, Sigma, Saint Quentin Fallavier Cedex, France) in 1 mL culture medium. After 24 h, the luciferase level was measured by luminescence. Cells were washed with PBS and centrifuged (7 min, 1500 rpm). After elimination of the supernatant, the homogenization buffer (0.2 mL of 8 mM MgCl_2_, 1 mM dithiotreitol, 1 mM Ethylenediaminetetraacetic acid (EDTA), 15% glycetol, 1% Triton X-100, 25 mM Tris-phosphate buffer pH 7.8) was poured into each well. The tissue culture plates were shaken and kept at 20 °C for 10 min. The solution was recovered and centrifuged (10 min, 3000 rpm). ATP (95 µL of a 2 mM solution of homogenization buffer without Triton-X100) was added to 60 µL of supernatant and vigorously mixed. Luminescence was recorded for 4 s using a luminometer (LUMAT LB 9501, Berthold, Wildbach, Germany) upon addition of 0.15 mL of 167 mM luciferin solution in water. The relative luciferase activity can be given to evaluate the inhibition of NFκB.

### 2.7. Assay Validation, Linearity of Calibration and Limit of Quantification (LOQ)

Calibration standards were prepared at five concentration levels from 50 to 200 µg·mL^−1^ for **1** and **2**, and from 5 to 50 µg·mL^−1^ for **3**. Every calibration standard was injected in triples, on three different days. The nine replicates were then pooled. The linearity of the response was evaluated by plotting calculated against theoretical concentrations to represent the linearity of the method; slopes appeared to be near to 1, and the intercept near to 0; *R*^2^ for **1**, **2** and **3** were evaluated at 0.9991, 0.9997 and 0.999, respectively. The calibration curves were estimated to be linear using this model, and the area of the peak corresponding to one compound was proportional to its concentration. The LOQ was defined as the lowest concentration on the calibration curve with a standard deviation in the tolerance domain of 5% for precision and accuracy. A LOQ of **1**, **2** and **3** was 10, 32 and 5 µg·mL^−1^, respectively.

### 2.8. Assay Validation—Precision

Intra-day precision was determined by assaying three replications of samples at five different concentrations levels. The results were used to construct the calibration curves. Inter-day precisions were assessed by assaying three standard mixtures freshly prepared once a day over three days (J1, J2, J4). The precision of the method was calculated as the relative standard deviation (RSD) of the concentration determined in all replicates. Intra-day and inter-day precision (RSD) were 0.16% and 0.24% for andrographolide (25 µg/mL), 0.85% and 0.68% for didehydroandrographolide (100 µg/mL) and 0.76% and 0.68% for neoandrographiside (20 µg/mL).

### 2.9. Assay Validation—Accuracy

Accuracy was determined by analyzing three replicates at 125, 100 and 20 µg/mL for **1**, **2** and **3**, respectively, once a day over three days. A fresh mixture was prepared each day. The accuracies were assessed by comparing the determined concentrations with the nominal concentrations. We found that the repeatability of the method was below 2% RSD and the accuracy of the method was 99.9%–101.5%, reliable with a tolerant deviation of 5%.

## 3. Results and Discussion

### 3.1. Effects of Isolated and Modified Compounds on TNFα Induced NFκB Transcriptional Activation

**Figure 5:**
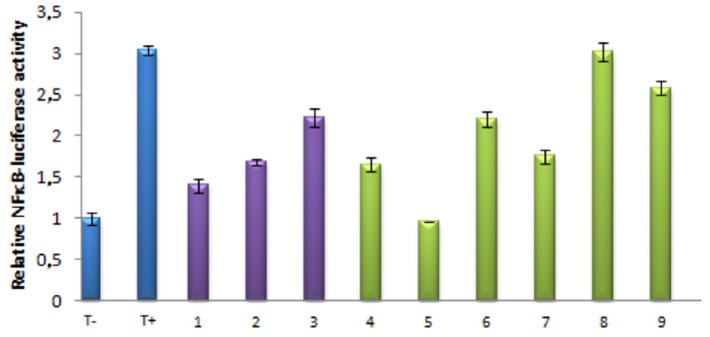
Relative NFκB-luciferase activity of pure compounds from **1** to **9**, 25 µg/mL.

Activation of A549 with TNFα increased NFκB transactivation luciferase activity and denoted as (T+). No activation by DMSO was reported as (T−). The samples were tested with a concentration of 25 µg/mL. The nine pure compounds and their inhibitory effects are shown in Figure 5. Inhibitory effects are in purple for the natural compounds (Figure 1), and in green for semisynthetic derivatives (Figure 4, Compounds **4** to **9**).

The results obtained for Compounds **4** and **7** showed that a modification of the diol present on the starting andrographolide did not change the anti-inflammatory activity. While, for Compounds **5**, **6**, **8** and **9**, results showed that the modification of the lactone (5-membered ring) could lead to a radical change in the anti-inflammatory activity. Indeed, Compound **8** did not significantly inhibit NFκB transcriptional activity, aldehyde **6** showed the same activity than neoandrographiside and Compound **5** showed the best inhibition of these nine derivatives.

In fact, previous experiments have shown that modification of the compound at the lactone level (5-membered ring system) can modify the anti-inflammatory activity of andrographolide. In order to understand the mechanism of inhibition of NFκB by andrographolide, andrographolide 1 was treated with NHBoc-L-cysteine methyl ester. A Modification of NFκB by site-directed mutagenesis [22] has shown that a cysteine residue would be involved in the formation of a covalent bond with andrographolide. We therefore put andrographolide in the presence of a cysteine residue, in a buffered medium approaching the biological environment, and we were able to isolate Compound **10**, resulting from the addition of cysteine on andrographolide, at 40% yield.

**Figure 6:**
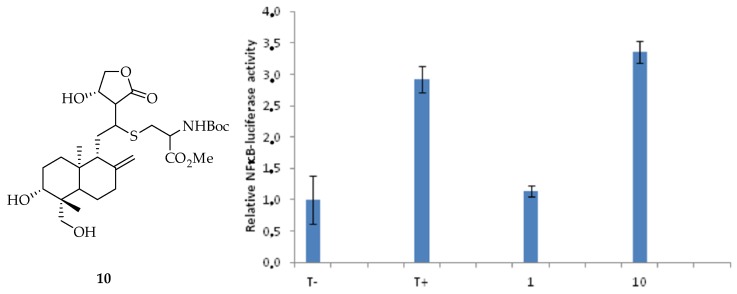
Structure of Compound **10** and relative NFκB-luciferase activity.

Compound **10** as shown in Figure 6 was isolated and tested for its biological effect. The results showed that this compound did not inhibit NFκB transcriptional activity yet. This means that andrographolide acts as a Michael acceptor in the inhibition of NFκB and that in Compound **10** the lack of inhibition was due either to the absence of the acceptor, or to the steric hindrance of the compound due to the addition of NFκB.

This last result could explain why Analogue **6** kept a significant inhibition. In this compound, addition of thiol is possible on carbonyl. Analogue **5** appeared to be the best Michael acceptor for the inhibition of NFκB transcriptional activity.

### 3.2. Quantitation of Commercial Preparations

As it has been shown that *Andrographis* extracts could have a better immunostimulant activity than andrographolide alone [32], we were interested in the difference of activity for mixtures with different ratios in andrographolide derivatives. Consequently, the quantification method was applied to four commercial preparations. The commercial samples were first extracted with methanol, as described in the Materials and Methods section. The extraction yields of dry substance were 44%, 40%, 17% and 33% for samples A to D, respectively. The four chromatograms were similar to those presented in Figure 3.

Concentrations determined by external standard quantitation method were presented in Table 1.

As expected, the analysis proved that the compositions of the commercial preparations are not the same. The dietary supplements B and C were reported to contain 10% *Andrographis* and it clearly appeared that both samples did not contain the same quantity neither in **1** nor in the rest of the compounds of the diterpenes family. Actually, B contains 10% andrographolide **1**, and C contains 10% of the three andrographolide derivatives **1**, **2** and **3**. Moreover, for the sample C, **1** is not the major compound. This analysis showed that there is a real difference in the labeling information of dietary supplements and the relative properties will not be the same.

### 3.3. Effects of the Extracts of Known Concentrations on TNFα Induced NFκB Transcriptional Activation

**Figure 7:**
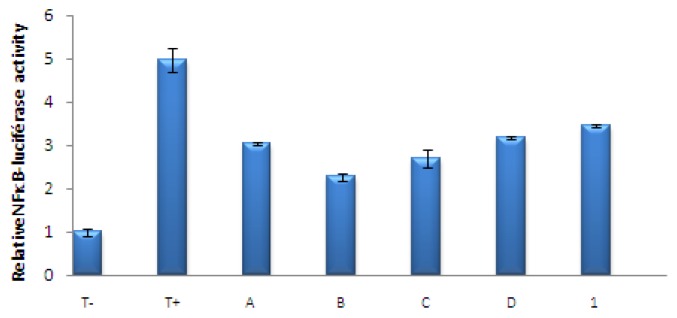
Relative NFκB-luciferase activity of A, B, C and D with 17 µg/mL andrographolide.

Using the quantitation of diterpenes in mixtures, we investigated the activity of herbal preparations incubating the same quantity of andrographolide, and we standardized the final concentration of andrographolide at 17 µg/mL. The results could show if there is a synergic or complementary effect of several compounds in the extracts for the inhibition of NFκB. The results are presented in Figure 7 and show that if the mixtures contain **1** as the major compound and only low amounts of **2** and **3** (as in A and D), the inhibitory activity follows the activity of Compound **1**. Nevertheless, for mixtures such as B and C where the quantity of **2** is not negligible, the inhibitory activity increases.

These results could explain those reported in the literature [31], showing that there were other compounds than andrographolide in *A. paniculata* extracts that act as an immunostimulant, and we proved herein that there was a complementary activity of **2** at the studied concentrations.

### 3.4. Characterization of Products **1**, **2** and **3**

Extracted compounds were characterized by standard spectral methods, NMR, mass Spectroscopy (ESIMS) and high resolution mass spectroscopy (HRMS), and conform to already-described data [33].

**1**: ^1^H NMR (DMSO, 400 MHz) δ 6.62 (1H, dt, J_11,12_ = 7.0 Hz, J_12,14_ 1.5 Hz, H12), 5.71 (1H, d, J_14,OH_ 6.0 Hz, OH14), 5.05 (1H, d, J_3,OH_ 5.0 Hz, OH-3), 4.91 (1H, t, J_14,15_ 6.0 Hz, H14), 4.81 (1H, s, H17A), 4.62 (1H, s, 17B), 4.39 (1H, dd, J_15A,15B_ 10Hz, J_14,15A_ 6.0 Hz, H15A), 4.13 (1H, dd, J_19B,OH_ 7.5 Hz, J_19A,OH_ 2.5 Hz, OH-19), 4.03 (1H, dd, J_15A,15B_ 10.0 Hz, J_14,15B_ 2.0 Hz, H15B), 3.84 (1H, dd, J_19A,19B_ 11.0 Hz, J_19A,OH_ 2.5 Hz, H19A), 3.29–3.20 (2H, m, H3, H19B), 2.46 (1H, m, H11A), 2.32 (1H, br d, J_7A,7B_ 13.0 Hz, H7A), 1.6–1.84 (2H, m, H7B, H11B), 1.75–1.61 (5H, m, H1A, H2, H6A, H9), 1.35 (1H, dq, J_6A,6B_ J_6B,7B_ 13.0 Hz, J_6B,7A_ 4.0 Hz, H6B), 1.23–1.17 (2H, H1B, H5), 1.08 (3H, s, H18), 0.66 (3H, s, H20). ^13^C NMR (DMSO, 100 MHz) δ 170.8 (C16), 148.5 (C8), 147.2 (C12), 129.9 (C13), 109.2 (C17), 79.3 (C3), 75.2 (C15), 65.4 (C14), 63.6 (C19), 56.4 (C9), 55.3 (C5), 43.1 (C10), 39.5 (C4), 38.4 (C7), 38.1 (C11), 37.4 (C1), 28.8 (C2), 24.8 (C6), 24.0 (C18), 15.6 (C20). ESIMS (negative mode) m/z 331 [M-H_2_O]^−^, 349 [M-H]^−^, 395 [M-H+HCOOH]^−^, (positive mode) 351 [M+H]^+^, 373 [M+Na]^+^. HRMS: calcd for C_20_H_31_O_5_ [M+H]^+^ 351.2167, found 351.2166.
**2**: ^1^H NMR (DMSO, 400 MHz) δ 7.65 (1H, brs, H14), 6.74 (1H, dd, J_11,12_ 15.5 Hz, J_9,11_ 10.0 Hz, H11), 6.12 (1H, d, J_11,12_ 15.5 Hz, H12), 5.03 (1H, d, J_3,OH_ 5.0 Hz, OH-3), 4,89 (2H, brs, H15), 4.73 (1H, s, H17A), 4.42 (1H, s, H17B), 4.12 (1H, t, J_OH,19B_ 6.5 Hz, OH-19), 3.84 (1H, dd, J_19A,19B_ 13.0 Hz, J_19A,OH_ 5.0 Hz, H19A), 3.28–3.16 (2H, m, H3, 19B), 2.36–2.32 (2H, m, H9, H7A), 1.97 (1H, m, H7B), 1.72 (1H, m, H2A), 1.58–1.54 (2H, m, H2, H6A), 1.40 (1H, dd, J_6A,6B_ 13.0 Hz, J_6B,7B_ 4.0 Hz, H6B), 1.28–1.12 (2H, m, H1), 1.07 (3H, s, H18), 0.76 (3H, s, H20). ^13^C NMR (DMSO, 100 MHz) δ 172.9 (C16), 149.5 (C8), 147.2 (C14), 134.7 (C11), 127.5 (C13), 121.6 (C12), 108.5 (C17), 79.1 (C3), 63.1 (C19), 61.0 (C9), 54.2 (C5), 42.8 (C4), 38.4 (C1), 36.7 (C7), 29.4 (C10), 28.1 (C2), 23.6 (C6), 23.4 (C18), 15.9 (C20). ESIMS (negative mode) m/z 331 [M-H]^−^, 377 [M-H+HCOOH]^−^, (positive mode) 355 [M+Na]^+^. HRMS: calcd for C_20_H_28_O_4_ [M+Na]^+^ 355.2035, found 355.2036.
**3**: ^1^H NMR (DMSO, 400 MHz) δ7.46 (1H, s, H14), 4.86–4.78 (6H, 3OH, H15, H17A), 4.59 (1H, s, H17B), 4.38 (1H, t, J_6′,OH_ 6.0 Hz, OH-6’), 4.02 (1H, d, J_1′,2′_ 8.0 Hz, 3.88 (1H, d, J_19A,19B_ 10.0 Hz, H19A), 3.63 (1H, dd, J_6′A,6′B_ 10.5 Hz, J_5′,6′A_ 6.0 Hz, H6A), 3.43 (1H, dd, J_6′A,6′B_ 10.5 Hz, J_5′,6′B_ 5.0 Hz, H6B), 3.17–3.04 (3H, m, H3’, H4’, H5’), 2.92 (1H, m, H2′), 2.36–0.90 (16H, m, H1, H2, H3, H5, H6, H7, H9, H11, H12), 0.95 (3H, s, H18), 0.61 (3H, s, H20). ^13^C NMR (DMSO, 100 MHz) δ 174.6 (C16), 148.1 (C8), 147.4 (C14), 132.6 (C13), 107.0 (C17), 104.0 (C1’), 77.3, 77.1, 70.6 (C3’, C4’, C5’), 74.0 (C2’), 71.2 (C15), 70.9 (C19), 61.5 (C6’), 56.1 (C9), 55.8 (C5), 38.8, 38.4, 36.1, 21.9, 19.0 (C1, C2, C3, C6, C7, C11, C12), 38.3 (C4), 28.0 (C18), 24.4 (C10), 15.6 (C20). ESIMS (negative mode) m/z 479 [M-H]^−^, 525 [M-H+HCOOH]^−^, (positive mode) 503 [M+Na]^+^. HRMS: calcd for C_26_H_41_O_8_ [M+H]^+^ 481.2795, found 481.2796.

## 4. Conclusions

The isolation of natural andrographolide derivatives allowed to modify their structure and to study the inhibition of NFκB transcriptional activity of pure analogues. Compound **5** highlighted the best anti-inflammatory activity from the nine tested derivatives. The HPLC quantitation method has been developed in order to quantify three major diterpenes in *A. paniculata* extracts. This method was applied for the quantitation of four commercial herbal preparations. The composition was compared to the inhibitory activity of NFκB of such preparations and showed that even if andrographolide **1** is the major and more potent active compound in the plant, the amount of didehydroandrographolide **2** is not negligible.

## Figures and Tables

**Table 1:** Quantitation of three diterpenes in commercial preparations. Concentrations given in µg·mL^−1^ ± SD.

Diterpene	A	B	C	D
**1**	176.7 ± 12	138.8 ± 0.2	82.0 ± 0.1	127.3 ± 0.3
**2**	95.5 ± 0.7	136.9 ± 3.6	96.1 ± 2.1	51.3 ± 0.3
**3**	18.3 ± 0.7	13.4 ± 0.4	26.4 ± 0.4	9.8 ± 0.3

## References

[1] Acharya R. (2017). A review of phytoconstituents and their pharmacological properties of Andrographis Paniculata (NEES). Int. J. Pharma Biol. Sci..

[2] Chowdhury A., Biswas S.K., Raihan S.Z., Das J., Paul S. (2012). Pharmacological Potentials of Andrographis paniculata: An Overview. Int. J. Pharmacol..

[3] Hossain M.S., Urbi Z., Sule A., Rahman K.M.H. (2014). *Andrographis paniculata* (Burm. f.) Wall. ex Nees: A Review of Ethnobotany, Phytochemistry, and Pharmacology. Sci. World J..

[4] Zhang L., Bao M., Liu B., Zhao H., Zhang Y., Ji X., Zhao N., Zhang C., He X., Yi J. (2019). Effect of Andrographolide and Its Analogs on Bacterial Infection: A Review. Pharmacology.

[5] Grainne H., Franco S., Antonio G., Diego C. (2019). Herbal medicinal products for inflammatory bowel disease: A focus on those assessed in double-blind randomised controlled trials. Phytother. Res..

[6] Rahman H., Kim M., Leung G., Green J.A., Katz S. (2017). Drug-Herb Interactions in the Elderly Patient with IBD: A Growing Concern. Curr. Treat. Options Gastroenterol..

[7] Farzaei M.H., Shahpiri Z., Bahramsoltani R., Moghaddamnia M., Najafi F., Rahimi R. (2017). Efficacy and Tolerability of Phytomedicines in Multiple Sclerosis Patients: A Review. CNS Drugs.

[8] Tan W.S.D., Liao W., Zhou S., Wong W.S.F. (2017). Is there a future for andrographolide to be an anti-inflammatory drug? Deciphering its major mechanisms of action. Biochem. Pharmacol..

[9] Majee C., Mazumder R., Choudhary A.N. (2019). Medicinal plants with anti-ulcer and hepatoprotective activity: A review. Int. J. Pharm. Sci. Res..

[10] Chua L.S. (2014). Review on Liver Inflammation and Antiinflammatory Activity of *Andrographis paniculata* for Hepatoprotection. Phytother. Res..

[11] Chao W.-W., Lin B.-F. (2012). Hepatoprotective Diterpenoids Isolated from *Andrographis paniculata*. Chin. Med..

[12] Lu J., Ma Y., Wu J., Huang H., Wang X., Chen Z., Chen J., He H., Huang C. (2019). A review for the neuroprotective effects of andrographolide in the central nervous system. Biomed. Pharmacother..

[13] Vijaya N.R., Abinaya R. (2019). An effect of cardioprotective activity in various medicinal plants-a review. Int. J. Curr. Pharm. Res..

[14] Rajendran H., Deepika S., Immanuel S.C. (2017). An Overview of Medicinal plants for Potential Cardio-Protective Activity. Res. J. Biotechnol..

[15] Shourie A., Chandwani A., Singh S., Rawal A., Vijayalakshmi U. (2018). Anticancerous potential of medicinal plants—A review. World J. Pharm. Res..

[16] Kumar M.S., Swati T., Archana S., Hyun O.S., Mook K.H. (2015). Andrographolide and analogues in cancer prevention. Front. Biosci. (Elite Ed.).

[17] Gorter M. (1911). The Bitter Constituent of Andrographis Paniculata Nees. Recueil des Travaux Chimiques des Pays-Bas.

[18] Jayakumar T., Hsieh C.-Y., Lee J.-J., Sheu J.-R. (2013). Experimental and Clinical Pharmacology of *Andrographis paniculata* and Its Major Bioactive Phytoconstituent Andrographolide. Evid. Based Complementary Altern. Med..

[19] Cava M.P., Chan W.R., Stein R.P., Willis C.R. (1965). Andrographolide: Further transformations and stereochemical evidence; the structure of isoandrographolide. Tetrahedron.

[20] Smith A.B., Toder B.H., Carroll P., Donohue J. (1982). Andrographolide: An X-ray crystallographic analysis. J. Crystallogr. Spectrosc. Res..

[21] Medforth C.J., Chang R.S., Chen G.-Q., Olmstead M.M., Smith K.M. (1990). A conformational study of diterpenoid lactones isolated from the Chinese medicinal herb *andrographis paniculata*. J. Chem. Soc. Perkin..

[22] Xia Y.F., Ye B.Q., Li Y.D., He X.J., Lin X., Yao X., Ma D., Slungaard A., Hebbel R.P., Key N.S. (2004). Andrographolide attenuates inflammation by inhibition of NF-kappa B activation through covalent modification of reduced cysteine 62 of p50. J. Immunol..

[23] Li W., Fitzloff J. (2002). HPLC with Evaporative Light Scattering Detection as a Tool to Distinguish Asian Ginseng (Panax ginseng) and North American Ginseng (Panax quinquefolius). J. Liq. Chromatogr. Relat. Technol..

[24] Xu L., Xiao D.-W., Lou S., Zou J.-J., Zhu Y.-B., Fan H.-W., Wang G.-J. (2009). A simple and sensitive HPLC–ESI-MS/MS method for the determination of andrographolide in human plasma. J. Chromatogr. B.

[25] Srivastava A., Misra H., Verma R., Gupta M. (2004). Chemical fingerprinting of *Andrographis paniculata* using HPLC, HPTLC and densitometry. Phytochem. Anal..

[26] Gu Y., Ma J., Liu Y., Chen B., Yao S. (2007). Determination of andrographolide in human plasma by high-performance liquid chromatography/mass spectrometry. J. Chromatogr. B.

[27] Chen L., Jin H., Ding L., Zhang H., Wang X., Wang Z., Li J., Qu C., Wang Y., Zhang H. (2007). On-line coupling of dynamic microwave-assisted extraction with high-performance liquid chromatography for determination of andrographolide and dehydroandrographolide in *Andrographis paniculata* Nees. J. Chromatogr. A.

[28] Ding L., Luo X.B., Tang F., Yuan J.B., Guo M., Yao S.Z. (2008). Quality control of medicinal herbs Fructus gardeniae, Common Andrographis Herb and their preparations for their active constituents by high-performance liquid chromatography–photodiode array detection–electrospray mass spectrometry. Talanta.

[29] Du Q., Jerz G., Winterhalter P. (2003). Separation of andrographolide and neoandrographolide from the leaves of *Andrographis paniculata* using high-speed counter-current chromatography. J. Chromatogr. A.

[30] Nanduri S., Nyavanandi V.K., Thunuguntla S.S.R., Velisoju M., Kasu S., Rajagopal S., Kumar R.A., Rajagopalan R., Iqbal J. (2004). Novel routes for the generation of structurally diverse labdane diterpenes from andrographolide. Tetrahedron Lett..

[31] Gomez-Bombarelli R., Calle E., Casado J. (2013). Mechanisms of Lactone Hydrolysis in Neutral and Alkaline Conditions. J. Org. Chem..

[32] Puri A., Saxena R., Saxena R.P., Saxena K.C., Srivastava V., Tandon J.S. (1993). Immunostimulant agents from *Andrographis Paniculata*. J. Nat. Prod..

[33] Villedieu-Percheron E. (2011). Study about an Anti-Inflammatory Natural Diterpenes Family. Ph.D. Thesis.

